# When Should I Start Planning For Retirement?: Comparing Perceptions of Retirement Planning Timelines

**DOI:** 10.1093/geroni/igaf122.1989

**Published:** 2025-12-31

**Authors:** Kathryn Warren, Adam Felts, Lisa D’Ambrosio, Joseph Coughlin

**Affiliations:** MIT AgeLab, Cambridge, Massachusetts, United States; Massachusetts Institute of Technology, Cambridge, Massachusetts, United States; Massachusetts Institute of Technology, Cambridge, Massachusetts, United States; Massachusetts Institute of Technology AgeLab, Cambridge, Massachusetts, United States

## Abstract

The demographic shift toward longer life expectancy changes how people are planning for retirement, necessitating both financial and lifestyle preparation for an increasing period of time beyond one’s career. The anticipated duration of life without employment income affects retirement savings goals and the length of working life, making early and strategic planning essential. To explore people’s attitudes toward the time necessary to prepare for retirement, the MIT AgeLab conducted a national online survey (N = 1152, ages 18-89), stratified by age and gender. Respondents were asked when they believed a person should begin to plan for life in retirement and when they expected they would (or, if already retired, when they did) retire. Among respondents who had not yet retired (N = 884), younger respondents anticipated a shorter preparation period. The 18-29 age group, on average, believed planning should begin 16.1 years before retirement, whereas those aged 60-69, still pre-retirement, reported an average preparation window of 30.0 years. These findings suggest that as individuals near their expected retirement age, their ideal timeframe for preparation lengthens. Similarly, retired respondents (N = 268) indicated that planning should begin at a younger age compared to their non-retired counterparts, reinforcing the importance of early preparation. This session will explore these shifting perceptions of retirement readiness, discuss potential psychological and behavioral factors influencing planning timelines, and highlight strategies for encouraging proactive financial and lifestyle planning across the lifespan.

